# Network Autoregressive Model for the Prediction of COVID-19 Considering the Disease Interaction in Neighboring Countries

**DOI:** 10.3390/e23101267

**Published:** 2021-09-28

**Authors:** Arash Sioofy Khoojine, Mahdi Shadabfar, Vahid Reza Hosseini, Hadi Kordestani

**Affiliations:** 1 Faculty of Economics and Business Administration, Yibin University, Yibin 644000, China; arashsioofy@yibinu.edu.cn; 2 Center for Infrastructure Sustainability and Resilience Research, Department of Civil Engineering, Sharif University of Technology, Tehran 145888-9694, Iran; 3 Institute for Advanced Study, Nanchang University, Nanchang 330031, China; v.r.hosseini@ncu.edu.cn; 4 Department of Civil Engineering, Qingdao University of Technology, Qingdao 266033, China; hadi@qut.edu.cn

**Keywords:** COVID-19, Iran timeseries prediction, infected cases, ARIMA model, correlation matrix, network autoregressive (NAR) model

## Abstract

Predicting the way diseases spread in different societies has been thus far documented as one of the most important tools for control strategies and policy-making during a pandemic. This study is to propose a network autoregressive (NAR) model to forecast the number of total currently infected cases with coronavirus disease 2019 (COVID-19) in Iran until the end of December 2021 in view of the disease interactions within the neighboring countries in the region. For this purpose, the COVID-19 data were initially collected for seven regional nations, including Iran, Turkey, Iraq, Azerbaijan, Armenia, Afghanistan, and Pakistan. Thenceforth, a network was established over these countries, and the correlation of the disease data was calculated. Upon introducing the main structure of the NAR model, a mathematical platform was subsequently provided to further incorporate the correlation matrix into the prediction process. In addition, the maximum likelihood estimation (MLE) was utilized to determine the model parameters and optimize the forecasting accuracy. Thereafter, the number of infected cases up to December 2021 in Iran was predicted by importing the correlation matrix into the NAR model formed to observe the impact of the disease interactions in the neighboring countries. In addition, the autoregressive integrated moving average (ARIMA) was used as a benchmark to compare and validate the NAR model outcomes. The results reveal that COVID-19 data in Iran have passed the fifth peak and continue on a downward trend to bring the number of total currently infected cases below 480,000 by the end of 2021. Additionally, 20%, 50%, 80% and 95% quantiles are provided along with the point estimation to model the uncertainty in the forecast.

## 1. Introduction

SARS-Cov-2 (COVID-19) is on the rise and it is quickly infecting new people every day. Currently, two years after the onset of this pandemic, this ascending trend has not yet stopped and it is even multiplying in some countries [1]. When a person is determined to be infected with the disease in a country, there may be two possibilities where he is infected:

1. The first case concerns the situation where both carriers and recipients of the disease are in the same country. This type of disease transmission is considered “local”;
2. The second is for cases infected in another country and transferred to a second country by travel. This type of disease transmission is called “imported”.

Communication among nations is one of the main causes of disease transmission, and is called disease interaction between countries in this paper. In addition to disease progress in target communities when examining its spreading profile, it is also of the utmost importance to reflect on its prevalence rate in other countries, including those with a high volume of travel [2]. The number of cases infected with this health condition can be thus deemed as a timeseries, taking account of the related statistics in the form of data over time [3].

In this regard, numerous researchers have thus far attempted to utilize a wide range of statistical tools to predict the number of cases of COVID-19 in the future to guide health care officials to make informed decisions [4]. For example, Shadabfar et al. used a susceptible–exposed–infected–vaccinated–recovered (SEIVR) model combined with the Monte Carlo (MC) sampling method to probabilistically investigate the COVID-19 spreading profile in the United States (USA) [5,6].

In general, different stochastic computations [7,8] and numerical methods [9,10,11,12,13,14] are exploited to assess the various aspects of the COVID-19 outbreak. In this sense, Katoch et al. used the autoregressive integrated moving average (ARIMA) model to forecast the COVID-19 dynamics in India [15]. Kumar Sahai et al. also modeled and predicted this pandemic via the ARIMA model [16]. Using the same ARIMA model, Malki et al. further predicted the second rebound of this disease; they also projected the end of the pandemic based on the ARIMA model [17]. Chaurasia et al. additionally used ARIMA and a regression model to forecast mortality rates in this respect [18]. Furthermore, Kumar et al. employed timeseries methods to analyze the COVID-19 spreading profile in ten affected countries [19]. Using the α-Sutte indicator and ARIMA, Attanayake et al. modeled COVID-19 [20]. Hernandez et al. correspondingly forecasted COVID-19 per region using the ARIMA model and polynomial functions [21]. Moreover, Yang et al. defined the data as timeseries and predicted the COVID-19 spreading profile in Wuhan, China [22].

Even though these studies have been to take advantage of different regression and optimization techniques to obtain the best fit of the data and consequently provide reliable timeseries forecasting, they typically suffer from one limitation, that is, their prediction remains independent of the disease spreading profile in other nations in the region. In fact, concerning the development trends of the disease interactions in neighboring countries, it seems ideal to measure the relationship between the disease spreading profiles in relevant nations to consider its impact on predicting the disease timeseries in target countries and regions.

To fill this gap, this paper utilizes a Network Autoregressive (NAR) Model. For this purpose, the COVID-19 data are initially retrieved from the World Health Organization (WHO) and the Johns Hopkins University online official websites and databases for seven different countries, namely, Iran, Turkey, Iraq, Azerbaijan, Armenia, Afghanistan, and Pakistan [23]. Thereafter, by constructing a network in the region, in which each vertex corresponds to a country and each edge represents the correlation of the total number of currently infected cases, the correlation matrix of the area is established. After that, the timeseries forecasting for Iran is performed using the NAR model, providing the number of infected cases up to December 2021. Comparing the root mean square error (RMSE) and mean absolute percentage error (MAPE) between autoregressive integrated moving average (ARIMA) and NAR models demonstrate that a better fit is obtained over the data once interactions among neighboring countries are taken into account. The method proposed in this paper can thus be implemented systematically to provide a reference for the investigation of the disease spreading profile in other countries and regions.

The rest of this study is organized as follows. Section 2 introduces the study area and then reviews the disease progression across the countries in the region concerned, from the onset of the COVID-19 pandemic in February 2020. Section 3 sheds light on the details of both methods implemented in this study, namely, the ARIMA and the NAR models, and subsequently describes how to consider the disease interactions in the neighboring nations in the proposed formulation. Next, in Section 4, the ARIMA and the NAR models are fitted to the existing data. In addition, upon comparing both methods, it is settled that the consideration of the disease interactions in the neighboring countries can enhance the prediction accuracy. Thus, the NAR model is employed to forecast the number of cases infected in Iran until the end of December 2021, and the results are reported. Then, in Section 5, the criteria for choosing the threshold are clarified in more detail. Finally, the contents are summarized and concluded in Section 6.

## 2. Target Region and Data Description

To implement this work, the records of the COVID-19 data from the WHO and Johns Hopkins University official websites are used [24]. It should be noted that the data reported by the WHO contain some uncertainty and do not reflect the complete and accurate status of the disease in society [25,26]. However, the approach presented in the current research is implemented based on the disease statistics provided by the WHO as the reference dataset. The authors do not claim that the prediction made in the paper is the real state of the disease in society but acknowledge that it will be the disease’s future according to WHO data. The data show confirmed cases, daily recovery, and death rates. The total of currently infected patients is accordingly calculated as follows:

(1) TotalCurrentlyInfected=TotalConfirmed−TotalRecovered−TotalDeath.

**Figure 1:**
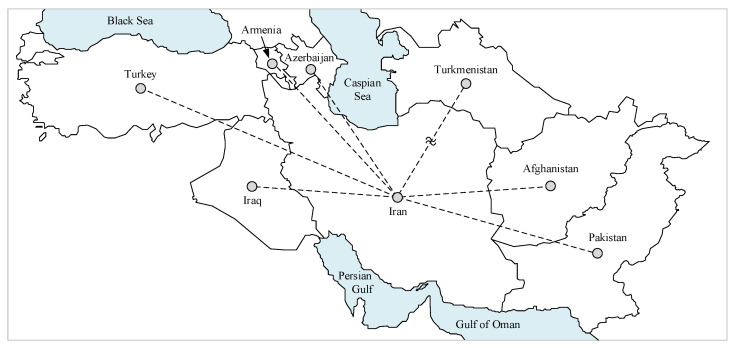
The region of interest investigated in the present study.

**Figure 2:**
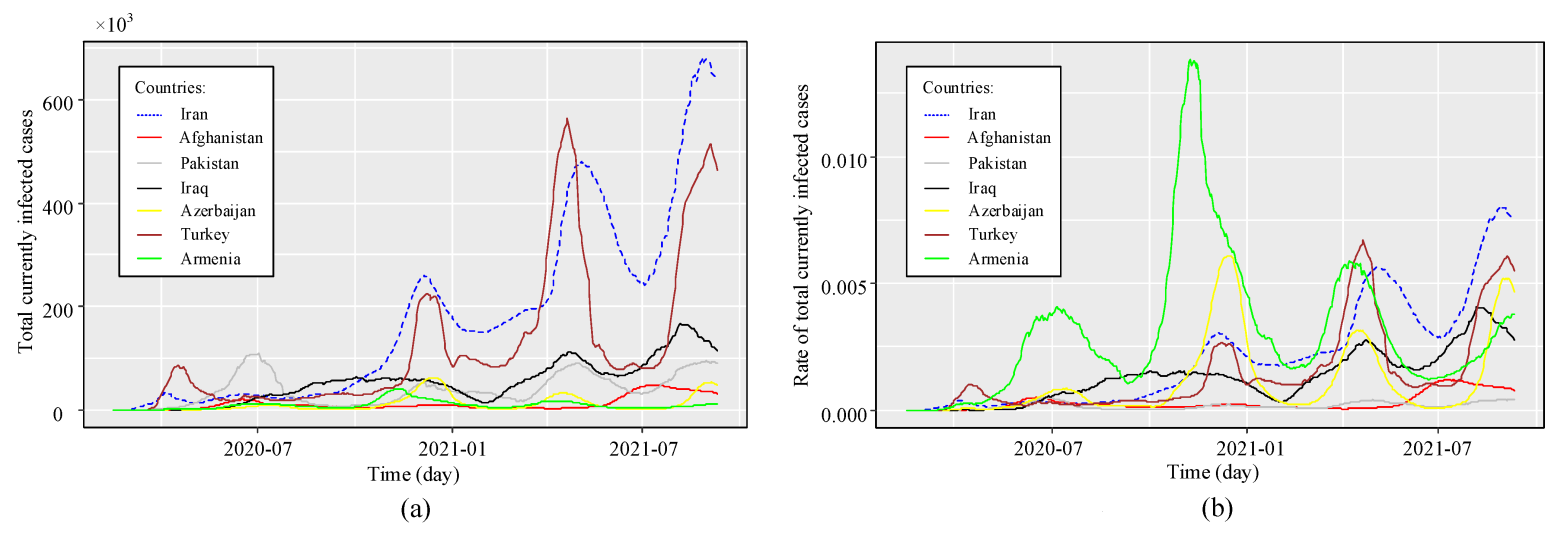
WHO COVID-19 data for seven different countries; (**a**) total currently infected cases, (**b**) rate of total currently infected cases.

As mentioned earlier, the primary purpose of this study is to address the impact of COVID-19 interactions in the neighboring countries on the timeseries forecasting model of the number of cases infected in Iran. As a result, some neighboring nations, including Turkey, Iraq, Azerbaijan, Armenia, Afghanistan, and Pakistan, are considered the target region here. The COVID-19 data from Turkmenistan are not publicly available, so they are not reflected in this study. A comparison of the geographic locations of these countries with Iran is further depicted in Figure 1. The timeseries of the rate of infected cases and infected cases in these nations as of 10 September 2021 are shown in Figure 2. A closer look at Figure 2 also reveals that different countries have so far experienced similar trends of this condition at the same time, which reinforces the hypothesis that the nations located in this region interact with the spread of the disease. For example, Iran and Turkey simultaneously experienced three peaks in March 2020, December 2020, and April 2021.

## 3. Model Formulation

### 3.1. NARI Model

In this section, the NAR model for total infected people, hereafter referred to as NARI, is provided, and its characteristics are explained. First, the data on the infected are transformed to a new timeseries, after that the correlations between countries are calculated and a network is created over the countries in which each edge denotes the correlation between a pair of countries. This network is then introduced into the NARI model to predict the time history of the target country given the correlation values. Assuming *N* is the number of countries, the difference of logs of the total infected people, $s_{it}$, is defined as follows:

(2) $$s_{it}=\delta \log\left(\frac{\mathrm{infected}\;(i,t)}{\mathrm{infected}\;(i,t-1)}\right),$$

where δ is a constant, which is a hyper parameter in the model. Using trial and error in the countries concerned, the optimal value of δ is computed as 0.5. Next, expanding this equation gives:

(3) $$s_{it}=\frac{1}{2}\log\left(\frac{\mathrm{infected}(i,t)}{\mathrm{infected}(i,t-1)}\right)=\log\left(\sqrt{\mathrm{infected}(i,t)}\right)-\log\left(\sqrt{\mathrm{infected}(i,t-1)}\right),$$

wherein infected(i,t) refers to the number of total infected cases from country *i* at time *t*. The NARI model is formulated as follows:

(4) $$s_{it}=\alpha_{0}+\alpha_{1}n_{i}^{-1}\sum_{j=1}^{N}a_{ij}s_{j(t-1)}+\alpha_{2}s_{i(t-1)}+\epsilon_{it},$$

where

(i) *N* is the number of countries;
(ii) $s_{it}$ represents the difference of logs of infected cases from country *i* at time *t*;
(iii) $A={\left(a_{ij}\right)}_{N\times N}$ shows the adjacency matrix of the correlation between the log-returns of *N* countries;
(iv) $n_{i}$ is the sum of the *i*th row at the adjacency matrix A;
(v) $\epsilon_{it}$ follows the normal distribution.

The assumption of a normal distribution for the error term in Equation (4) has also been adopted in other studies such as [27]. The main idea behind assuming a normal distribution for the noise term is that with this assumption we have the smallest variance between all of the estimators. This assumption helps the algorithm to approximate the MLE in a straightforward process and to facilitate the time prediction process. For more details, refer to [28].

The cross-correlations between the infected cases in different countries are similarly considered in terms of matrix C, whose elements are given through the following equation [29]:

(5) $$c_{ij}\equiv \frac{\langle s_{i}s_{j}\rangle -\langle s_{i}\rangle \langle s_{j}\rangle }{\sqrt{\left(\langle s_{i}^{2}\rangle -{\langle s_{i}\rangle }^{2}\right)\left(\langle s_{j}^{2}\rangle -{\langle s_{j}\rangle }^{2}\right)}},$$

where the brackets mean the temporal average over the infected cases during the time considered. Then, $c_{ij}$ can vary between [−1,1]. The case of $c_{ij}=1\left(-1\right)$ also denotes that two countries *i* and *j* are completely correlated (anti-correlated), while $c_{ij}=0$ implies that they are uncorrelated.

Suppose that $A={\left(a_{ij}\right)}_{\left(N\times N\right)}$ is the adjacency matrix of the correlations among *N* countries; by adjusting a threshold as θ, −1≤θ≤1, the matrix A is defined as follows [30]:

(6) $$a_{ij}=\begin{array}{cc} 1 & if\;i\neq j\;\mathrm{and}\;c_{ij}\geq \theta \\ 0 & otherwise, \end{array}$$

and $n_{i}=\sum_{j=1}^{N}a_{ij}$ refers to the *i*th row sum of the adjacency matrix, and $E(\epsilon_{it})=0$ and $var\left(\epsilon_{it}\right)=\sigma^{2}$. This threshold value and its calculation details are described in Section 5. For convenience, Equation (4) can be rewritten in a matrix form as:

(7) $$\begin{array}{c} {\left(s_{1t},\ldots ,s_{Nt}\right)}^{⊺}={\left(1,\ldots ,1\right)}^{⊺}\alpha_{0}+\alpha_{1}\left(\begin{array}{ccccc} \frac{1}{n_{1}} & 0 & \cdots & 0 & \\ 0 & \frac{1}{n_{2}} & \cdots & 0\\ \vdots & & \ddots & 0\\ 0 & \cdots & 0 & \frac{1}{n_{N}} \end{array}\right)A+\alpha_{2}\left(\begin{array}{ccccc} 1 & 0 & \cdots & 0 & \\ 0 & 1 & \cdots & 0\\ \vdots & & \ddots & 0\\ 0 & \cdots & 0 & 1 \end{array}\right) \end{array}\;+\left(s_{1(t-1)},\ldots ,s_{N(t-1)}\right)+{\left(\epsilon_{1t},\ldots ,\epsilon_{Nt}\right)}^{⊺},$$

or in a concise form as:

(8) $$S_{t}=A_{0}+GS_{t-1}+\varepsilon_{St},$$

in which $S_{t}=\left(s_{1t},\ldots ,s_{Nt}\right)\in R^{N}$, $A_{0}=\alpha 1$ wherein $1={\left(1,\ldots ,1\right)}^{⊺}$, $G=\alpha_{1}W+\alpha_{2}I$ in which $W=diag\{n_{1}^{-1},\ldots ,n_{N}^{-1}\}A$, and I is an identity matrix.

Under the NARI model framework, the model might be based on three factors; first, $s_{it}$ might be affected by itself but from the previous time point, $s_{i(t-1)}$, called the autoregressive effect; second, $s_{it}$ might be influenced by its neighbors, which are collected by $\left\{j:a_{ij}=1\right\}$, labeled the “neighborhood effect”. The unexplained variation should also be attributed to an independent random noise, $\epsilon_{it}$. For example, for a country i=1 at t=3, $s_{13}$ is as follows:

(9) $$\begin{array}{cc} s_{13} & =\alpha_{0}+\underset{\mathrm{neighborhoods}\;\mathrm{effect}}{\underset{︸}{\alpha_{1}n_{1}^{-1}\sum_{j=1}^{N}a_{1j}s_{j2}}}+\overset{\mathrm{authoregresive}\;\mathrm{effect}}{\overset{︷}{\alpha_{2}s_{12}}}+\underset{\mathrm{independent}\;\mathrm{noise}}{\underset{︸}{\epsilon_{13}}}\\ \; & =\alpha_{0}+\alpha_{1}\frac{a_{12}s_{22}+\cdots +a_{1N}s_{N2}}{a_{12}+\cdots +a_{1N}}+\alpha_{2}s_{12}+\epsilon_{13}. \end{array}$$

Therefore, $\alpha_{2}s_{i(t-1)}$ is not incorporated into the first term. It can be proved that $S_{t}$ has a stationary property (for more details see [31]). To estimate $\alpha =(\alpha_{0},\alpha_{1},\alpha_{2})$, maximum likelihood estimation (MLE) is also used as follows:

(10) $$min_{\alpha }∥S_{t}-A_{0}-GS_{t-1}∥.$$

For estimating the unknown parameter α, the NARI model (Equation (4)) is rewritten as:

(11) $$s_{it}=\alpha_{0}+\alpha_{1}w_{i}^{⊤}S_{t-1}+\alpha_{2}s_{i(t-1)}+\epsilon_{it}.$$

Then, it is written as:

(12) $$s_{it}=Y_{i(t-1)}^{⊤}\alpha +\epsilon_{it},$$

where $Y_{i(t-1)}={\left(1,w_{i}^{⊤}S_{t-1},s_{i(t-1)}\right)}^{⊤}$ and $w_{i}={\left(a_{ij}/n_{i}:1\leq j\leq N\right)}^{⊤}$ indicate the *i*th row vector of *W*. Suppose $Y_{t}={\left(Y_{1t},\ldots ,Y_{Nt}\right)}^{⊤}$, then the above model can be written as:

(13) $$S_{t}=Y_{t-1}\alpha +\varepsilon_{t}.$$

Then, a maximum likelihood (ML) estimator in the logarithmic form can be obtained as follows:

(14) $$\begin{array}{cc} L(\alpha ,\sigma^{2}) & =-\frac{N}{2}\ln2\pi -\frac{N}{2}\ln\sigma^{2}-\frac{1}{2\sigma^{2}}{\left(\sum_{t=1}^{T}S_{t}-\sum_{t=1}^{T}Y_{t-1}\alpha \right)}^{⊤}\left(\sum_{t=1}^{T}S_{t}-\sum_{t=1}^{T}Y_{t-1}\alpha \right). \end{array}$$

Differentiating this expression with respect to α, the ML estimates will be as follows:

(15) $$\begin{array}{cc} \frac{\partial L}{\partial \alpha^{⊤}} & =-\frac{1}{2\sigma^{2}}(-2\sum_{t=1}^{T}Y_{t-1}^{⊤}S_{t}+2\sum_{t=1}^{T}Y_{t-1}^{⊤}Y_{t-1}\alpha )=0. \end{array}$$

Therefore,

(16) $$\hat{\alpha }={\left(\sum_{t=1}^{T}Y_{t-1}^{⊤}Y_{t-1}\right)}^{-1}\sum_{t=1}^{T}Y_{t-1}^{⊤}S_{t}.$$

Substituting Equation (13) into the estimator $\hat{\alpha }$ in Equation (16), there is:

(17) $$\hat{\alpha }=\alpha +{\left(\sum_{t=1}^{T}Y_{t-1}^{⊤}Y_{t-1}\right)}^{-1}\sum_{t=1}^{T}Y_{t-1}^{⊤}\epsilon_{St}.$$

### 3.2. ARIMA Model

Box and Jenkins [32] published a technique to merge both autoregressive (AR) and moving average (MA) models, called the ARMA (*p*, *q*) model, as a union of AR (*p*) and MA (*q*) models, generally deployed for univariate timeseries modeling. The ARMA (*p*, *q*) model is thus presented as follows:

(18) $$Y_{t}=c+\varepsilon_{t}+\sum_{i=1}^{p}\varphi_{i}X_{t-i}+\sum_{i=1}^{q}\theta_{i}\varepsilon_{t-i},$$

where the $\theta_{1},\ldots ,\theta_{q}$ and $\varphi_{1},\ldots ,\varphi_{p}$, are the parameters of the model and ε is the white noise. If the series is not stationary at the first level, there is a need to subtract it by *d*(d=1,2,3,…) times to make it stationary. Such a timeseries model is called an ARIMA (*p*, *d*, *q*) model.

There are three steps in ARIMA model creation, namely identification, parameter estimation, and diagnostic checking [33,34]. In this regard, for the identification process of the model, after checking the stationarity of the timeseries, the AR and MA terms are derived from the auto-correlation function (ACF) plot. ACF is a statistical metric of the correlation that is used to check if previous values in the timeseries analysis have a certain relationship with the latest values or not [35]. After that, ARIMA parameters, namely (p,d,q), are estimated by the least square method. The three main methods commonly used to select appropriate models are Akaike’s Information Criterion (AIC), the Bayesian Information Criterion (BIC) and the Second-order Akaike’s Information Criterion (AICc), which are presented in Equations (19)–(21) for AIC, BIC and AICc, respectively [32,36].

(19) AIC=−2log(L)+2k=−2log(L)+2(p+q+P+Q)

(20) BIC=−2log(L)+kln(n)=−2log(L)+(p+q+P+Q)ln(n)

(21) AICc=−2log(L)+2k(n/(n−k−1)),

where *n* refers to the size of the series and *k* denotes the number of the parameters of the ARIMA method. It is experimentally proved that the given model becomes efficient when the AIC value is smaller. According to [34], an optimal forecast model is selected based on the best fitting that has the minimum AIC value of the group.

To evaluate the prediction models, the following statistical measures are used for i(1,…,7) as follows:

(22) $$\mathrm{RMSE}\left(i\right)=\sqrt{\frac{1}{T}\sum_{t=1}^{T}{\left(s_{it}-{\hat{s}}_{it}\right)}^{2}}$$

(23) $$\mathrm{MAPE}\left(i\right)=\frac{100}{T}\sum_{t=1}^{T}\left|\frac{s_{it}-{\hat{s}}_{it}}{s_{it}}\right|,$$

where $s_{it}$ denotes the actual value and ${\hat{s}}_{it}$ and T are the modeled values and the total number of days.

## 4. Implement the Model for Each Country in the Region

### 4.1. ARIMA Model Results

**Figure 3:**
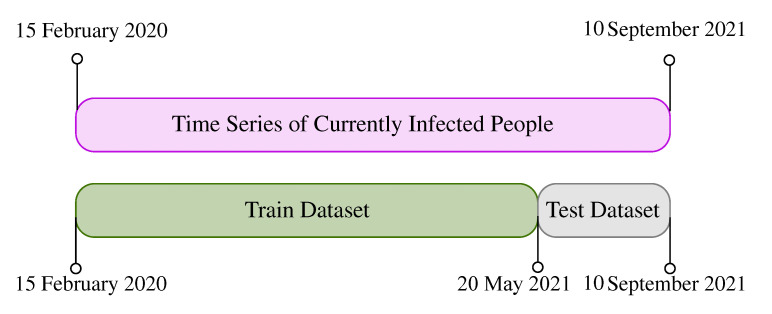
Timetable of train and test datasets.

The ARIMA model is used in this study as a benchmark to compare the results with the proposed NAR method. To implement the ARIMA model on it, the data are split from 15 February 2020 to 10 September 2021 into two parts; the first part, the training dataset, from 15 February 2020 to 20 May 2021, and the second part, the testing dataset, from 21 May 2021 to 10 September 2021. This data division process is applied to the COVID-19 data of all countries. Therefore, it is shown once in Figure 3. Considering the training part, the ARIMA model is conducted; then, the results are validated with the testing dataset once the parameters are estimated.

After splitting the data into the training and test sets, they are transformed to $s_{it}$ for smoothing purposes as expressed in Equation (2). If the data are found to be non-stationary for each country; they are made so by subtracting them from the previous day. The number of times the timeseries data become stationary through difference disposal becomes the value of parameter *d*.

Upon stabilizing the data, the parameters are estimated. First, the ACF of the *d*-order difference timeseries is calculated. The order of the Auto-correlation Function (ACF) exceeding the confidence boundary lag also becomes the value of *q*. Second, the value of *p* is computed, which is the order of the Partial Auto-correlation Function (PACF) exceeding the confidence boundary lag. PACF gives the partial correlation of a stationary timeseries with its own lagged values. By observing the ACF and PACF of the residuals, it is determined whether they are white noise or not. Consequently, the fit of the model is assessed by checking the $R^{2}$ value. Ultimately, the model is validated and evaluated by applying the ARIMA method to predict the remaining 10% of the data. After that, the RMSE is used, as explained in Section 2, to evaluate the model. The whole process is depicted in Algorithm 1.
**Algorithm 1:** The procedure of modeling using ARIMA.
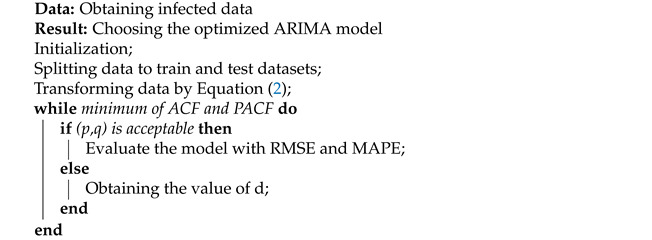


**Figure 4:**
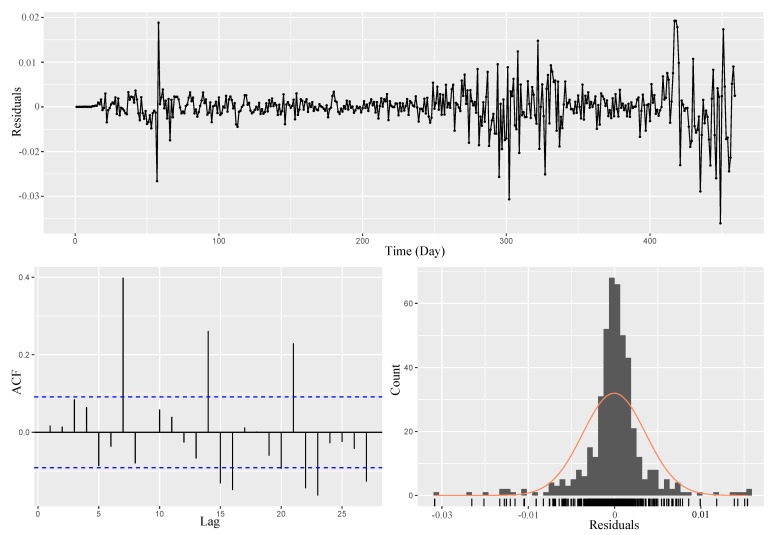
Residuals from ARIMA (2, 2, 2), Iran.

The ARIMA models are fit for the datasets of each country to compare them with the proposed model. Both Augmented Dickey–Fuller (ADF) and Kwiatkowski–Phillips–Schmidt–Shin (KPSS) tests also authenticate that the training timeseries are stationary at a 5% significance level. Accordingly, there is a need to apply the differencing method two times for Iran’s dataset. Later, diverse models are designed by adjusting various parameters for the MA and AR components of the ARIMA model, as summarized in Table 1. The ARIMA (2,2,2) model additionally assumes AICc criteria. Therefore, the chosen model is checked for some assumptions. The residual analysis of the model is presented in Figure 4. The Ljung–Box test on the residuals, as well as the squared residuals, is also statistically significant at a 5% level with *p*-value =0.9153. Therefore, the selected model with the minimum summary measures is appropriate with the lowest AIC, BIC, and AICc values.

**Figure A1:**
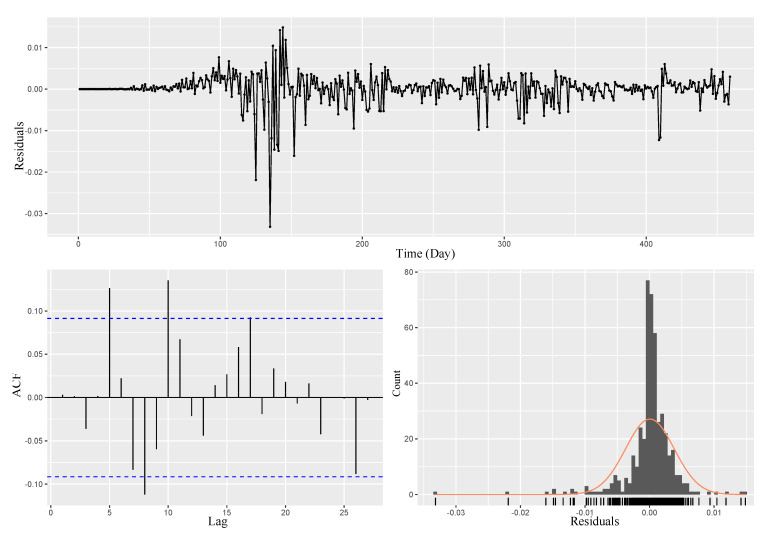
Residuals from ARIMA (2, 1, 3), Afghanistan.

**Figure A2:**
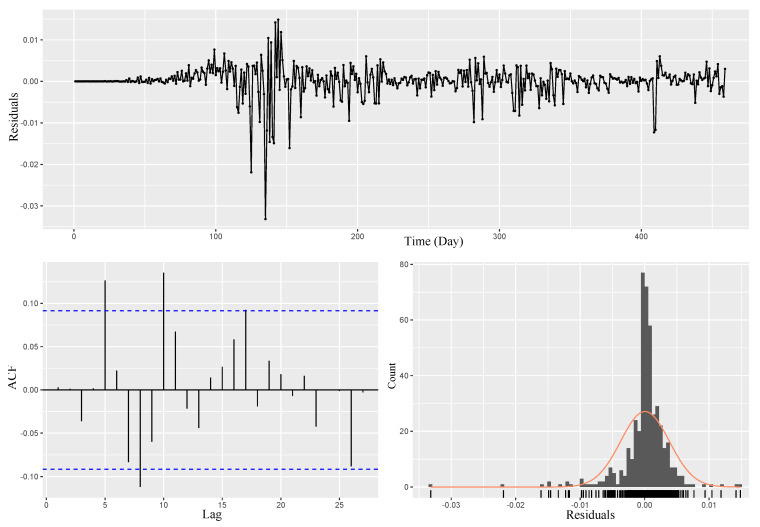
Residuals from ARIMA (2, 1, 3), Pakistan.

**Figure A3:**
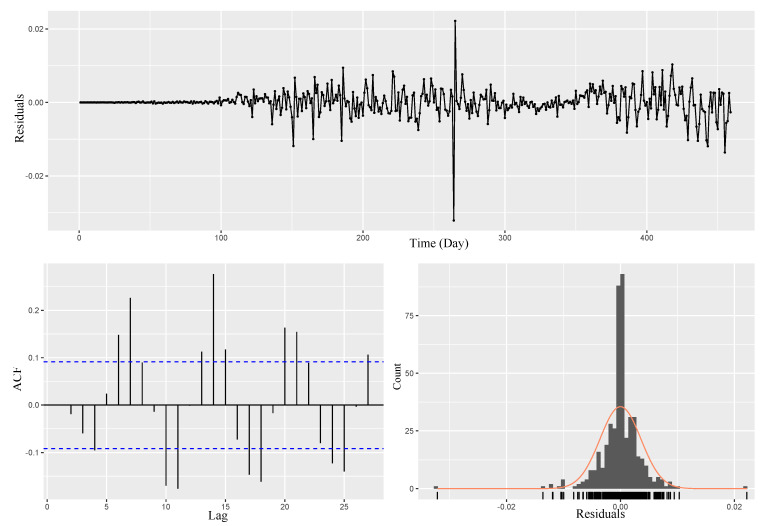
Residuals from ARIMA (1, 1, 3), Iraq.

**Figure A4:**
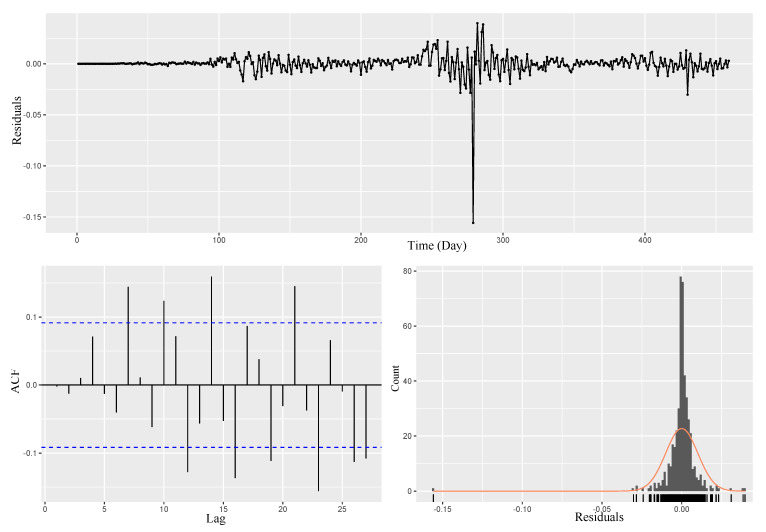
Residuals from ARIMA (5, 1, 4), Armenia.

**Figure A5:**
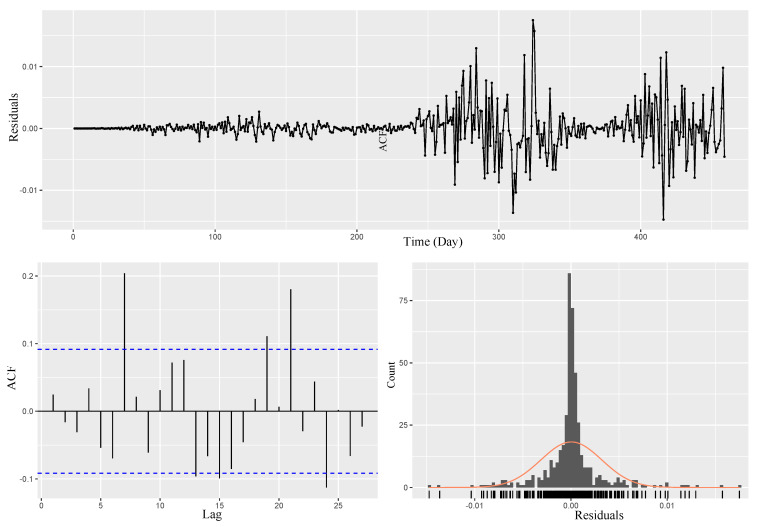
Residuals from ARIMA (2, 1, 2), Azerbaijan.

**Figure A6:**
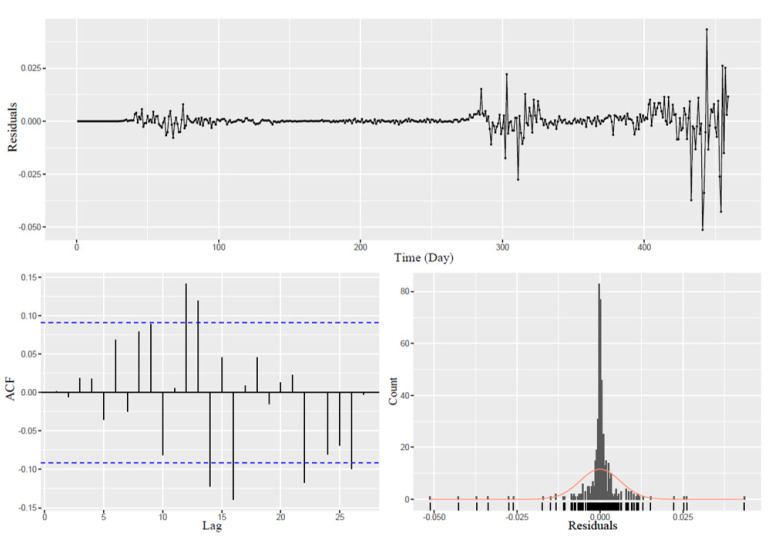
Residuals from ARIMA (4, 1, 1), Turkey.

The same approach is implemented in the training data series of other countries. The best model for each country is also selected, which has minimum AIC, BIC, and AICc measures, whose results are summarized in Table 2. The residual analysis of the best model in each country is depicted in Figure A1, Figure A2, Figure A3, Figure A4, Figure A5 and Figure A6 in the Appendix A.

### 4.2. NARI Model Results

**Figure 5:**
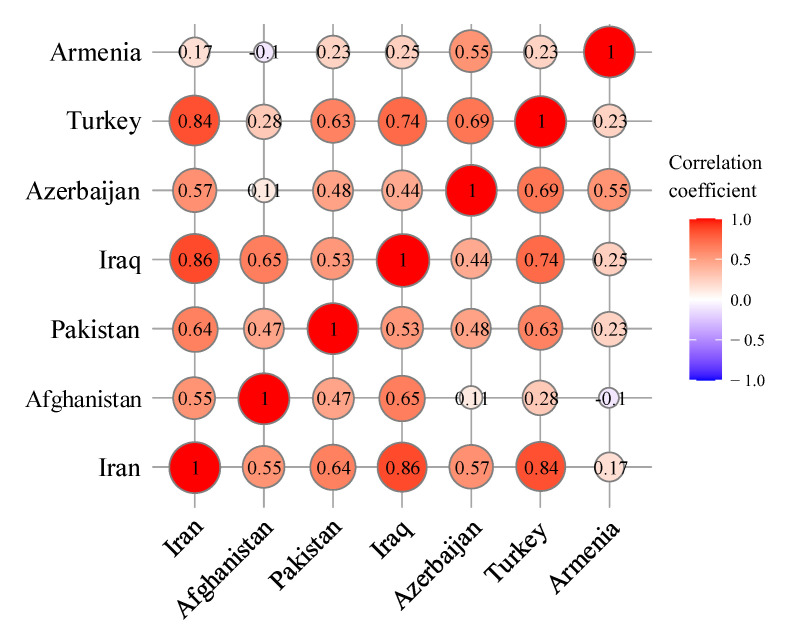
Correlation among countries.

In this section, the constructed NARI model is deployed on the COVID-19 dataset. As mentioned in the previous section, the data are split into two subsets of training and test datasets, as shown in Figure 3. The NARI model is then applied to the training dataset of each country. For this purpose, the training dataset is first transformed into a smooth timeseries, calculated by Equation (2). Then, the correlation matrix of countries is created by Equation (5), as reported in Figure 5. This allows the NARI model to be established for the whole network using Equation (4), given the parameter α as α=(0.014,0.005,0.79). The overall process of estimating α is presented in Algorithm 2.
**Algorithm 2:** The procedure of modeling using NARI.
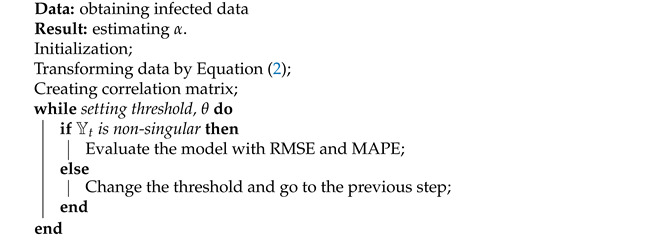


Upon estimating the parameter θ, the constructed NARI model needs to be validated. For this purpose, the 50 days are predicted using the constructed ARIMA models in the previous section and are then compared with the 50 days of the test dataset through the RMSE and MAPE metrics. The same procedure is also repeated for the NARI model, and the results are subsequently compared. The outcomes for both models are summarized in Table 3, wherein the NARI model outperforms the ARIMA one.

**Figure 6:**
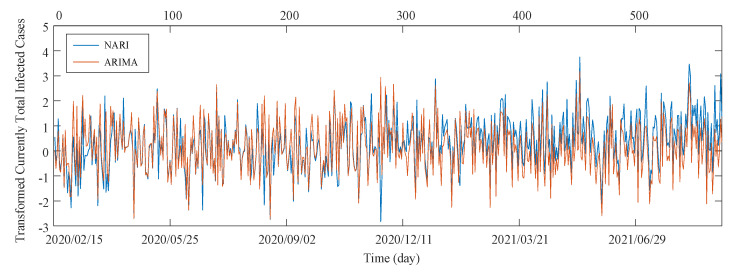
NARI model and ARIMA model for transformed currently total infected cases in Iran.

**Figure 7:**
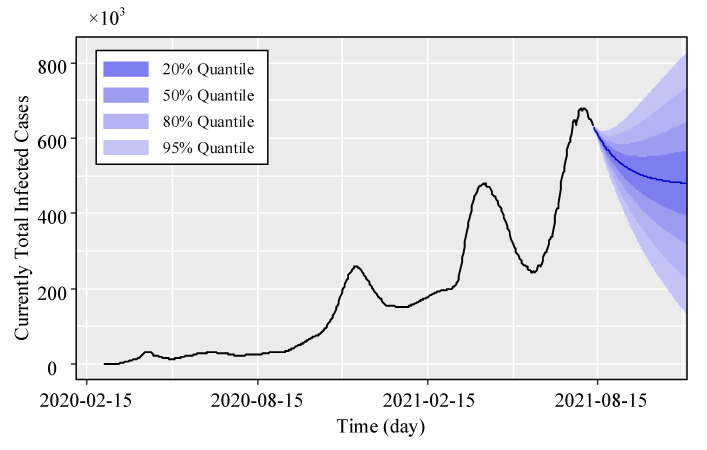
Forecast of currently total infected cases in Iran for 110 days.

The graphs of the transformed data of the total infected cases in Iran and the modeled data using the NARI and ARIMA models are shown in Figure 6. Upon finding the best COVID-19 modeling, the next 110 days of the disease are predicted according to the infected cases. The forecast results are plotted in Figure 7.

Timeseries forecasting for the spreading disease profile can be implemented considering disease interactions in different countries or without any correlation among them. Using the NARI model, considering these interactions, helps the constructed model to examine one of the most critical characteristics of disease transmission between societies and significantly enhances the accuracy of the timeseries prediction. This issue can be assessed by comparing the two models of ARIMA and NARI. It is shown in Table 3 that the RMSE for Iran, considering the disease interactions among neighboring countries, equals 3.06 for the NARI model and without considering it equals 5.42 for the ARIMA model. This means that the deliberation of the disease communications in neighboring countries promotes the prediction certainty substantially. Therefore, more reliable determinations can be made by policymakers to control the disease. It is of note that this prediction is based on the current pandemic situation. The results will also be altered in the case of events that change the present circumstances, such as enforcing stricter social distancing or wider public vaccination.

The forecast shows that the disease trend in Iran has passed the fifth peak, and the downward trend of the disease will continue after September 2021, so that the total number of infected cases per day will fall to less than 480,000 by the end of 2021. This is the point estimation prediction obtained from the NARI model, which is the most statistically possible case. However, Figure 7 also provides the quantiles of 20%, 50%, 80% and 95%, indicating the prediction uncertainty. The lower bounds of the 20%, 50%, 80% and 95% quantiles, respectively, indicate that the downward trend could be relatively more severe, bringing the total number of infected cases below 390,000, 320,000, 220,000 and 130,000 by the end of 2021. In addition, the upper limits of these quantiles indicate a possible new peak in Iran’s new COVID-19 data. In this case, the total number of infected cases corresponding to 20%, 50%, 80% and 95% quantiles may reach 560,000, 640,000, 730,000 and 820,000, respectively, by the end of 2021.

## 5. Discussion

In Section 3, it was discussed that the adjacency matrix, representing the disease interaction among nations, is formed by adopting a threshold and comparing it to the correlation matrix. In order to determine this threshold and explain how to implement the process, additional analysis is required, which is discussed in this section.

To explain the approach adopted to compute the correlation threshold, it should be explained that there are two constraints to meet. First, none of the $n_{i}$ in Equation (7) should be zero; otherwise, an infinity term would appear in this equation. Besides, a value of $n_{i}$, which can minimize the RMSE, is preferred as it helps the algorithm gain better accuracy. To implement an algorithm that can satisfy these two conditions, the threshold value is defined as a decision variable. An external loop is then added to the main algorithm to change the value of θ and calculate the corresponding $n_{i}$ and RMSE. The results for different θ values from 0 to 1 with an increment of Δθ=0.1 are reported in Table 4.

As seen, θ values greater than 0.6 give infinity values for $n_{i}$, thus cannot be selected as a correlation threshold in the algorithm. Moreover, for the rest of the cases, θ=0.5 gives the minimum amount of RMSE. Hence, it is selected as the optimal case and is utilized in the model implementation.

## 6. Summary and Conclusions

In this paper, the COVID-19 spreading profile in Iran is predicted in view of the influence of the severity and correlation of the disease in neighboring countries. To this end, the timeseries of COVID-19 infection among seven countries in the region, including Iran, Turkey, Iraq, Azerbaijan, Armenia, Afghanistan, and Pakistan, are downloaded from the online databases provided by the WHO and Johns Hopkins University. Then, a network is formed in the region to establish the correlation matrix among the countries concerned. Furthermore, by incorporating the correlation matrix into the proposed formula and calculating the model coefficients, the NARI model is used to predict the number of infected cases in Iran up until the end of September 2021, taking into account the impact of the disease in neighboring countries. The main results obtained in this study are as follows:

1. The correlation matrix obtained from the network of the countries in the region shows that the greatest impact of COVID-19 on Iran comes from Iraq, Turkey, Pakistan, Azerbaijan, Afghanistan and Armenia, with correlation coefficients of 0.86, 0.83, 0.64, 0.56, 0.55, 0.16, respectively. This result can also be seen in the trend of infected cases. The increasing/decreasing trend and the number of disease peaks in Iran, Iraq, and Turkey are very similar and have occurred within a short period of time. This indicates that the proposed correlation criterion is able to capture the similarity between infected data and disease peaks;
2. Timeseries predictions can be made with or without considering disease interactions in different countries. Incorporating the disease interaction not only helps the algorithm assess one of the most important components of disease transmission between societies but also significantly increases the accuracy of the timeseries prediction. This issue can be examined by comparing the two models of ARIMA and NARI. The RMSE with and without considering the disease interactions among neighboring countries is equal to 5.42 and 3.06 for ARIMA and NARI, respectively. This means that the consideration of the disease interactions in neighboring countries improves the prediction accuracy considerably. As the model’s accuracy in predicting disease increases, more reliable tools are provided for policymakers to take informed controlling decisions;
3. The point estimation obtained from the NARI model indicates that the number of infected cases in Iran declines after September 2021, so the total currently infected cases will fall below 480,000 by the end of 2021. According to the prediction corresponding to the lower limit of 20%, 50%, 80%, and 95% quantiles, the total number of infected persons will fall below 390,000, 320,000, 220,000 and 130,000, respectively, by the end of 2021.

Iran’s close neighbors, sharing common borders, and their impacts on the COVID-19 spreading profile in Iran are examined in this paper. However, ideally, more distant countries in the region that have direct or indirect demographic relationships with Iran can be also considered. Such a high volume of interactions between the countries requires the construction of a larger network to cover more countries and to subsequently provide a more reliable prediction. Such a model imposes more complexities on the problem, making the prediction results more accurate and reliable. Moreover, various factors, such as hospitalization, social distancing, quarantine, and so forth, can affect the number of people infected with COVID-19 in a society. However, the spreading profile of disease under the effects of the involved factors is not in the scope of the current research. Simulating the disease spread, taking into account the factors involved, requires establishing a system of differential equations in a so-called compartmental model and solving it incrementally to simulate the disease profile in the future. This topic is under investigation by the authors.

## Figures and Tables

**Table 1:** Summary measures for AICc in ARIMA model candidates—Iran series.

Model	AICc
ARIMA (2, 2, 2)	7510.893
ARIMA (0, 2, 0)	7568.916
ARIMA (1, 2, 0)	7568.436
ARIMA (0, 2, 1)	7565.978
ARIMA (1, 2, 2)	7561.546
ARIMA (2, 2, 1)	7561.854
ARIMA (3, 2, 2)	7566.861
ARIMA (2, 2, 3)	7541.272
ARIMA (1, 2, 1)	7563.57
ARIMA (1, 2, 3)	7563.364
ARIMA (3, 2, 1)	7564.825
ARIMA (3, 2, 3)	7512.12

**Table 2:** The results of ARIMA orders and residual analysis.

Country	(*p*, *d*, *q*)	AIC	AICc	BIC
Azerbaijan	(2,1,2)	3959.84	3959.71	3939.21
Afghanistan	(2,1,3)	3797.35	3797.17	3772.59
Pakistan	(2,1,3)	3797.35	3797.17	3772.59
Turkey	(4,1,1)	3358.17	3357.98	3333.41
Armenia	(5,1,4)	2886.98	2886.48	2845.71
Iraq	(1,1,3)	3959.84	3959.71	3939.21

**Table 3:** RMSE and MAPE metrics of ARIMA and NARI models for 7 countries.

		Iran	Afghanistan	Pakistan	Iraq	Armenia	Azerbaijan	Turkey
ARIMA model	RMSE	5.42	1.82	4.20	3.04	3.85	3.01	10.1
	MAPE	8.21	0.68	3.33	2.43	4.1	3.53	3.93
NARI model	RMSE	3.06	1.02	1.75	1.41	1.09	1.96	8.33
	MAPE	2.15	0.42	2.87	1.01	1.81	1.55	1.85

**Table 4:** Different correlation threshold and corresponding $n_{i}$ and RMSE.

θ	$n_{1}$	$n_{2}$	$n_{3}$	$n_{4}$	$n_{5}$	$n_{6}$	$n_{7}$	RMSE
0.1	7	6	7	7	7	7	6	3.98
0.2	5	4	6	6	5	6	4	3.85
0.3	5	3	5	5	5	4	1	3.64
0.4	5	3	5	5	5	4	1	3.64
0.5	5	2	3	4	3	4	1	3.06
0.6	3	1	2	3	1	4	0	NaN
0.7	2	0	0	2	0	2	0	NaN
0.8	2	0	0	1	0	1	0	NaN
0.9	0	0	0	0	0	0	0	NaN

## Data Availability

Data, models, or code that support the findings of this study are available from the authors upon reasonable request.

## Appendix A

This section presents the residuals obtained from the ARIMA model of six countries, including Afghanistan, Pakistan, Iraq, Armenia, Azerbaijan, and Turkey. As discussed in Section 4.1, the results of these graphs are used to find the optimized ARIMA model for the number of infected people in the tracked countries. Further details of the calculations and modeling process of these graphs can be found in Section 4.
